# Using Digital Technologies in Clinical HIV Research: Real-World Applications and Considerations for Future Work

**DOI:** 10.2196/jmir.7513

**Published:** 2017-07-31

**Authors:** Jessica Andriesen, Sheana Bull, Janan Dietrich, Jessica E Haberer, Barbara Van Der Pol, Yegor Voronin, Kristin M Wall, Christopher Whalen, Frances Priddy

**Affiliations:** ^1^ Fred Hutchinson Cancer Research Center Seattle, WA United States; ^2^ Colorado School of Public Health Denver, CO United States; ^3^ Perinatal HIV Research Unit (PHRU) Faculty of Health Sciences University of the Witwatersrand Soweto South Africa; ^4^ Massachusetts General Hospital/Harvard Medical School Boston, MA United States; ^5^ University of Alabama at Birmingham Birmingham, AL United States; ^6^ Global HIV Vaccine Enterprise New York, NY United States; ^7^ Emory University Atlanta, GA United States; ^8^ Research Data & Communications Technologies Corp. Garrett Park, MD United States; ^9^ International AIDS Vaccine Initiative New York, NY United States

**Keywords:** clinical trial, HIV, mobile phone, text messaging, biometric identification, observational study privacy, data collection

## Abstract

**Background:**

Digital technologies, especially if used in novel ways, provide a number of potential advantages to clinical research in trials related to human immunodeficiency virus (HIV) and acquired immune deficiency syndrome (AIDS) and may greatly facilitate operations as well as data collection and analysis. These technologies may even allow answering questions that are not answerable with older technologies. However, they come with a variety of potential concerns for both the participants and the trial sponsors. The exact challenges and means for alleviation depend on the technology and on the population in which it is deployed, and the rapidly changing landscape of digital technologies presents a challenge for creating future-proof guidelines for technology application.

**Objective:**

The aim of this study was to identify and summarize some common themes that are frequently encountered by researchers in this context and highlight those that should be carefully considered before making a decision to include these technologies in their research.

**Methods:**

In April 2016, the Global HIV Vaccine Enterprise surveyed the field for research groups with recent experience in novel applications of digital technologies in HIV clinical research and convened these groups for a 1-day meeting. Real-world uses of various technologies were presented and discussed by 46 attendees, most of whom were researchers involved in the design and conduct of clinical trials of biomedical HIV prevention and treatment approaches. After the meeting, a small group of organizers reviewed the presentations and feedback obtained during the meeting and categorized various lessons-learned to identify common themes. A group of 9 experts developed a draft summary of the findings that was circulated via email to all 46 attendees for review. Taking into account the feedback received, the group finalized the considerations that are presented here.

**Results:**

Meeting presenters and attendees discussed the many successful applications of digital technologies to improve research outcomes, such as those for recruitment and enrollment, participant identification, informed consent, data collection, data quality, and protocol or treatment adherence. These discussions also revealed unintended consequence of technology usage, including risks to study participants and risks to study integrity.

**Conclusions:**

Key lessons learned from these discussions included the need to thoroughly evaluate systems to be used, the idea that early success may not be sustained throughout the study, that some failures will occur, and considerations for study-provided devices. Additionally, taking these key lessons into account, the group generated recommendations on how to move forward with the use of technology in HIV vaccine and biomedical prevention trials.

## Introduction

Those who sponsor, conduct, and analyze data from clinical trials actively seek new digital tools and technologies that could reduce costs or timelines; potentially improve clinical operational efficiencies; facilitate recruitment and retention of volunteers; and improve data collection, quality, and analysis [1]. However, deployment of such tools is not always straightforward and may have implications for regulatory approval, ethics reviews, statistical analysis, study design, and other aspects of clinical trial conduct. Moreover, practical experience with a given technology often uncovers benefits and pitfalls that were not anticipated at the planning stage. The use of such technologies in trials related to human immunodeficiency virus (HIV) and acquired immune deficiency syndrome (AIDS) poses unique challenges due to stigmatization of HIV in many communities [2], which may decrease participants’ willingness to share information with study staff. Therefore, it is critical to review past experiences and to discuss innovative uses of digital technologies that could be adopted in future clinical trials.

## Methods

In April 2016, the Global HIV Vaccine Enterprise surveyed the field for research groups with recent experience in novel applications of digital technologies in HIV clinical research and convened these groups for a 1-day meeting. Real-world uses of various technologies were presented and discussed by 46 attendees, most of whom were researchers involved in the design and conduct of clinical trials of biomedical HIV prevention and treatment approaches. After the meeting, a small group of organizers reviewed the presentations and feedback obtained during the meeting and categorized various lessons-learned to identify common themes. A group of 9 experts developed a draft summary of the findings that was circulated via email to all 46 attendees for review. Taking into account the feedback received, the group finalized the considerations that are presented here.

Many of the listed considerations are not specific to HIV and AIDS research but potentially can be applied to clinical studies of other diseases. However, due to the group’s focus on HIV and AIDS, we aimed to highlight unique aspects of dealing with this disease and did not fully evaluate the relevance of these considerations to other diseases.

The rapidly changing landscape of digital technologies presents a challenge for creating future-proof guidelines for technology application. To address this concern, the group included experts with many years of experience in applying digital technologies to clinical research, who were able to point to long-term trends in the industry and identify concerns, summarized below, that are likely to persist in the foreseeable future.

## Results

### Successful Applications of Digital Technologies to Improve Research Outcomes

#### Recruitment and Enrollment

**Figure 1:**
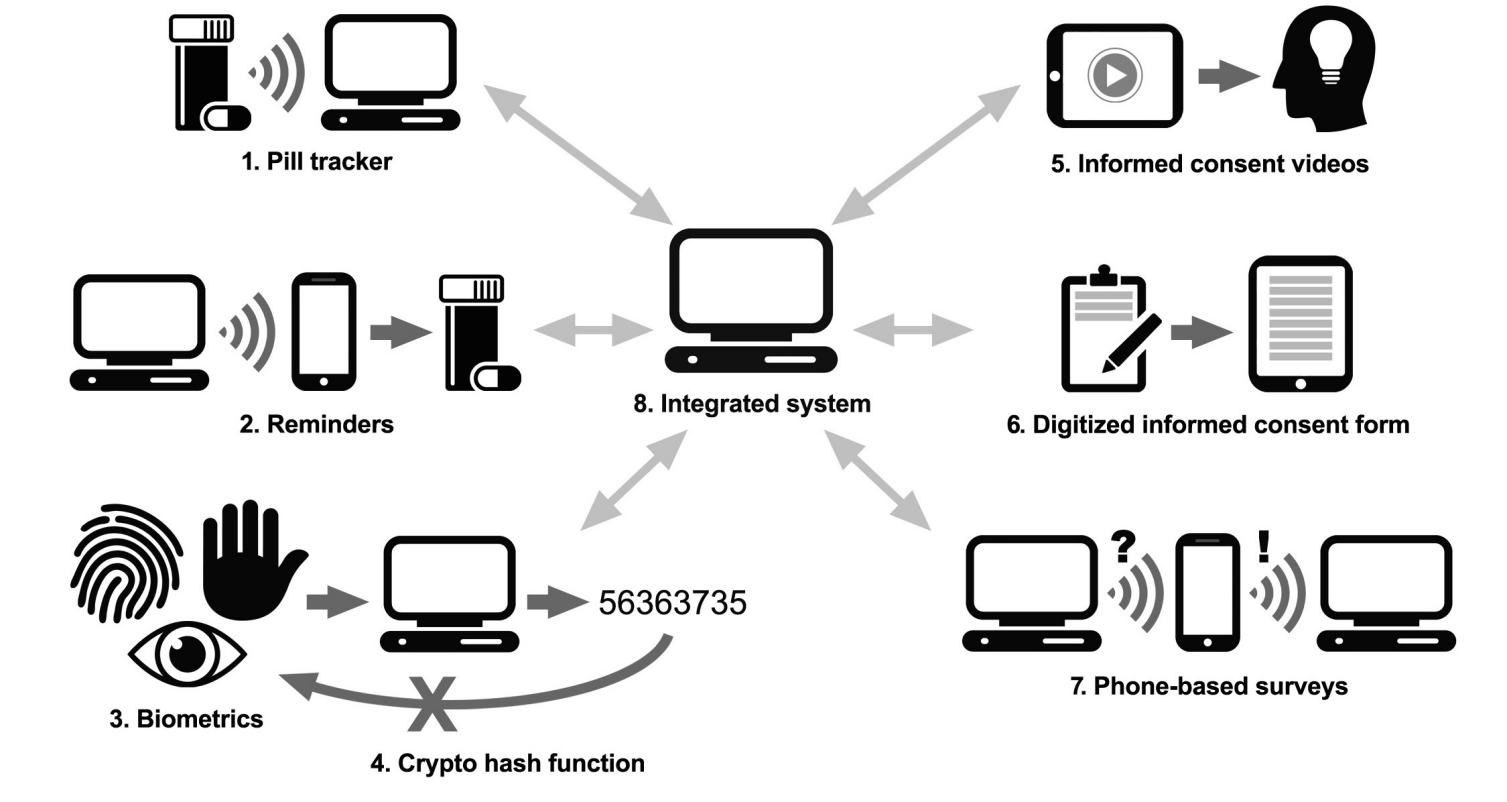
Examples of digital technologies.

When conducting HIV-related research, it is critical to be able to recruit from populations that have been previously underrepresented in research. The advent of digital technologies offers new opportunities for the recruitment and enrollment of these populations. Technologies necessary for research surveillance and other forms of data collection are increasingly becoming common in low- and middle-income settings [3,4] and have facilitated recruitment and enrollment of diverse populations in the United States [5]. For example, use of mobile devices such as tablets and phones for data collection within and outside the clinic increases the convenience of participation in research studies and positively influences participant response rates [6]. Using algorithms to tailor recruitment messages to individuals (eg, by sending personalized letters, emails, or text messages [short message service, SMS]) also increases response rates [7]. Figure 1 shows examples of these digital technologies, including: (1) electronic systems embedded in pill bottles which allow recording of every instance of bottle opening, providing researchers with a verifiable record of adherence; (2) messages sent via cell phones, which can be used to improve adherence to drugs, encourage specific behaviors, or remind about appointments; (3) various biometric measures (fingerprints, palm prints, and iris scans being the most common) which can be used to identify participants; (4) cryptographic hash functions that allow for one-way matching of participants, protecting their privacy in situations when trial records may be compromised; (5) videos or interactive forms that can be used to improve participants’ understanding by providing more visual information by allowing self-pacing, or by ensuring comprehension before allowing progress to following sections; (6) scanning and electronic storage of documents such as informed consent forms to improve record keeping; (7) cell phones, which can be used to reduce the need for in-person visits or to gather information from participants in real time, reducing recall bias; and (8) some systems which integrate two or more of the digital technologies allowing more efficient project management and better data integration.

#### Participant Identification

Devices such as electronic fingerprinting technologies, retinal scans, or palm identification can be used to identify participant coenrollment in different trials within or across trial sites, as well as “fraudulent” participation (occurring when a nonenrolled individual appears for a trial visit, presumably to receive trial incentives)[8,9]. In the context of community-, clinic-, or other cluster-randomized trials, they can help identify and possibly prevent intervention contamination, which occurs when participants in one group or cluster migrate into another group and “share” the intervention in a control group. For example, in a community-randomized trial of safe sex messaging when people interact with members of a control community, they may share the messages they have heard. This would result in moving the evaluation measures of the two communities closer and masking the true effect size [10].

Assessing participant identification in a longitudinal fashion can be used to prevent such issues, which not only compromise study and data integrity but also may harm patients (eg, if coenrolled patients receive double doses of a vaccine or other experimental product or if noneligible participants receive a vaccine or other experimental product)[8,9,11]. When these issues cannot be prevented, biometric identification used in longitudinal fashion can help measure the magnitude of such contamination and mitigate this effect during analysis [12-14].

Several studies indicate that the use of biometric devices is feasible in an array of resource settings and acceptable among both research study staff and participants [15-17]; in the case of some stigmatized populations, there is even data suggesting that biometric identifiers may be preferred over providing one’s name or other identification during study visits [18].

Biometric tools, if integrated into the study with strong cryptography, can provide a means of protecting participant identity while providing even greater levels of identity assurance than the traditional tools [19]. For example, systems that use retina scans convert the unique image of the participant’s eye into digital information using a cryptographic hash (an algorithm that links data to a string of symbols that cannot be used to identify the original image). That key is only available to operators of the study making identification of a participant with only the image of their eye virtually impossible. This approach significantly reduces the potential for release of data to the wrong participant or if there is a compromise of the study data that would breach confidentiality. In addition, assurance of identity also reduces the risk that the participant is misidentified and therefore receives an incorrect regimen.

#### Informed Consent

Videos and interactive electronic systems can provide alternatives to traditional informed consent procedures. Whereas data are mixed on whether video-based informed consent offers improvement over the traditional paper documentation of informed consent, there are indications that people may experience greater comprehension of the research process with video-based consent [20]. This may be especially helpful in populations where literacy rates are a concern. Additionally, interactive programs or websites can offer user-centered approaches to informed consent that include simpler layouts and opportunities for self-pacing that can help to ensure participants comprehend study processes [20].

#### Data Collection

Digital technologies have made study conduct more efficient as well. Systems such as the Research Electronic Data Capture (RedCap) [15,16], Open Data Kit [17], and MagPi [21] offer researchers the capacity to streamline the workflow and collect data more efficiently. These systems combine storage of participant information, validation of informed consent, and in some cases reminder recalls and communication with participants in a single program, which simplifies project management and removes the need for posthoc integration of information from multiple sources [16,22]. This technology in turn improves the workflow so that research coordinators, and data collectors can use a single system to understand who has enrolled, who needs to complete informed consent, and who needs to complete assessments or return for follow up.

#### Data Quality

Digital data collection for psychosocial characteristics of participants offers important advantages. Automated skip patterns, also known as conditional branch logic, present the participant with questions that are customized based on their responses to previous questions. For example, if a participant indicates sexual encounters in the past week, more questions appear to gather additional details about those encounters, but these questions are omitted if the participant answers that there was no sexual activity [6,22]. Forced item response (when participants are required to answer a question before they can move to the next question)—offering a “don’t want to answer” option to avoid coercion—helps reduce or eliminate missing data, substantially contributing to data quality [6,23,24]. Advances in technologies for measurement of biomedical variables can help improve integration of biomedical measures with psychosocial measures in a single dataset [25].

#### Protocol or Treatment Adherence

Clinical trials are typically designed to determine the efficacy of a medication or other biomedical intervention. True biologic efficacy, however, can only be determined in the setting of high adherence [26]. Incomplete adherence reduces the trial’s power to determine if suboptimal efficacy is due to the intervention or to participants’ behavior. The importance of this relationship between adherence and efficacy has been highlighted in the recent clinical trials assessing pre-exposure prophylaxis (PrEP) against HIV infection [27].

Numerous methods exist for measuring adherence, including self-report, pill or product counts, pharmacy refill records, drug levels, and electronic adherence monitoring (eg, pill or product bottles that record openings). Each method has its pros and cons, and none is considered a gold standard [28]. For example, self-reported adherence is often an overestimate because of social desirability bias (ie, wanting to report high adherence to please a clinician or researcher regardless of the true adherence behavior) and recall bias (ie, not accurately remembering forgotten doses). Digital technologies can be used to help optimize these measures in several ways.

##### Cellular Phone–Based Adherence Support

Given the near ubiquity of cellular phones, even in low-income countries, multiple studies have explored the use of short message service (SMS) and interactive voice response to potentially improve upon self-reported data [29,30]. Some data suggest that the relative anonymity of a cellular phone (compared with an in-person interview) leads to reduced social desirability bias [31]. Moreover, the convenience of cellular-based data collection allows for more frequent data collection, thus reducing recall time. However, validation of cellular phone-based reporting is limited [32], and further research into this measurement approach is needed.

##### Real-Time Adherence Monitoring Devices

Electronic adherence monitors provide a day-to-day assessment of pill or product container openings, which can be a highly informative, objective measure of adherence behavior. Whereas this technology has existed for many years, wireless versions have become available in recent years, enabling the use of this type of adherence data in real-time in both resource-rich and resource-limited regions [33,34]. Real-time electronic monitoring devices can be used both for measurement and for intervention at the precise time when nonadherence is occurring. Three recent trials assessing real-time adherence linked to SMS reminders revealed improvements in adherence, although the extent of the impact was variable [35-37]. Real-time adherence monitoring has yet to be used in a large-scale HIV clinical trial but has great potential to improve adherence and increase the discrimination between biological and behavioral aspects of efficacy.

### Unintended Consequences of the Use of Technology

#### Risks to Study Participants

##### Data Security as a Risk to Study Participants

For clinical trial participants, the data security practices of the researchers and their sponsors are critically important. For participants in HIV research trials, these practices are even more critical as breaches of confidentiality may negatively impact participants’ lives [38,39]. These negative impacts could include concerns around physical safety due to breach of location information or could result in social stigma of assumed infection status for participants and their family members.

Digital technologies used in clinical research offer opportunities to improve the data security whereas also introducing vulnerabilities for the breach of that data. Some examples of how the new technologies offer improvements for the security of the participant data include encryption for data within storage systems, encryption of the data transmission stream from one system to another, and the ability to use identity assurance tools such as biometrics that verify the participants’ identity without maintaining personally identifiable information in the study management systems [40].

Simultaneously, new technologies are providing unexpected ways that the data security can be breached. Location services on mobile phones record the location and use of apps and connected devices such as accelerometers and biosensors, which are already used in clinical protocols [41]. These location data can be shared inadvertently by other applications, in metadata of other files, or by the phone itself. Updates to applications or the phone operating system can change the way location data or storage is handled by the device without the knowledge of data managers or study managers.

Additionally, the use of wearable technologies, which often include new data gathering and transmission tools, presents new opportunities for unexpected data release [42]. Many of these systems are consumer- and vendor-optimized and not designed for use in clinical studies. Commercial companies often have an interest in collecting and monetizing personal information, not protecting it.

##### Social Impacts to Study Participants

As noted above, cellular technology and electronic adherence monitors have great potential for adherence measurement and intervention; however, ethical concerns may arise in using this technology, primarily around privacy [43].

Participants may be able to utilize mobile phones to capture sensitive behavioral data in real time and in a confidential manner; however, the use of a study-provided phone may pose a risk to those participants who do not disclose study participation to their partner, family members, or roommates. As participants may be requested to keep a study-provided phone with them at all times in order to submit the daily survey records, participants may inadvertently disclose their study participation. Moreover, the participant may be accused of cheating on a partner or hiding a secret if he or she is discovered to be using a secret “second” mobile phone.

Risks are also present when participants use their own phones for study communication. Cellular phones in developing countries are commonly shared by multiple people [44], and an SMS sent to a trial participant may be seen by others, disclosing HIV status or trial participation. Protections against such situations include the use of nondescript messages that only the intended recipient will recognize, a personal identification number (PIN) to trigger an SMS with trial-specific content, and flash SMS or unstructured supplementary service data (USSD) protocols (ie, the messages disappear once read). Although such protections may not be needed in all contexts and some individuals may prefer direct messages [45], privacy should be considered when designing the use of SMS in any clinical research.

Electronic adherence monitors may attract attention in some settings such as rural villages or in stigmatized populations such as injection drug users. This attention may lead to unintended disclosure of HIV status or trial participation. The degree of concern expressed by participants in research studies has ranged from low to moderate [46,47]. To protect against this situation, users of electronic adherence monitors should plan to store, carry, and use them in an inconspicuous manner. Device manufactures can make alerts for missed doses (eg, lights or audible notifications) optional. In the future, use of these devices in clinical care may reduce concerns about privacy; however, they are currently limited to the research context.

Implementation of these and other technologies requires a careful understanding of the specific population(s) using them and the concerns they may have. Participant counseling may also be used to reduce concerns of inadvertent information release (eg, encouraging HIV status disclosure when it is safe to do so). Interestingly, at least one study found that an electronic adherence monitor was used to start discussions of disclosure [48].

#### Risks to Study Integrity

##### Introduction of Bias

The use of skip patterns may have unintended consequences for data collection [49]. Bias in responses to digital questionnaires can occur because of the length of mobile or Web-based surveys conducted at specific time points in a study. For example, a recent study of adolescent male sexual behaviors used mobile phone daily diaries that varied in length based on the number of partners reported by the respondents [49]. Participants reporting no sexual partners answered a total of 38 questions, whereas those reporting the maximum of 5 partners could see a total of 748 items. As a result, there was a tendency among participants to report fewer partners than they actually had in order to reduce the amount of time spent completing the daily diaries.

One way to address this is to limit the number of highly detailed follow-up items triggered by a “yes” response to sexual behavior and to add alternative, general information items to “no” responses so that the entire diary has a smaller number of questions and that gaming the system by responding “no” does not shorten the length of the diary.

##### Participation Fatigue

As with any traditional data collection method, participation fatigue can occur with electronic data capture methods such as daily diaries or periodic surveys. Participants with children and a very busy working and school schedule may find it especially difficult to complete study procedures daily as part of their busy day schedules. Therefore, if daily participation is required, then a shorter duration of study participation is recommended. If the study duration is longer, then active engagement of participants would be recommended to sustain motivation, such as lotteries [50,51]. Alternatively, study procedures could be completed based on particular events or on an ad hoc basis to maintain the novelty of study participation.

The issue of participation fatigue presents a particular concern given the power of electronic and Web-based data capture. Investigators now have the capacity to collect nearly limitless amounts of highly specific and often sensitive data using these tools. This desire to “measure everything” needs to be mitigated with the understanding that patient fatigue issues have not been alleviated as compared with traditional data collection methods. Therefore, investigators need to be thoughtful about the questionnaire design including effective skip patterns and innovative incentives and thoroughly pilot test new instruments before final implementation. As with any type of behavioral data collection, the key is to focus on the necessary variables that aid accomplishment of the protocol objectives and not gather more data than one can or will analyze.

## Discussion

### Key Lessons Learned From Early Technology Implementations

#### Systems Need to Be Thoroughly Evaluated

##### Staff Training

Thought and care need to be put into the setup and maintenance of the system to be used, including assessment of the need for training of site staff. For example, biometric identification technologies often require training to ensure high-quality capture of biometric measures, especially in limited resource settings [11]. This can be addressed by developing didactic and practical trainings, job aids, error logs, and scheduling intermittent refresher trainings.

##### System Testing

Additionally, the feasibility and use of proposed technology should be piloted on site prior to trial launch with study staff and potential participants. For example, technologies that are Internet-dependent will face issues when implemented in low-resource settings, particularly in rural areas. Whereas mobile Internet coverage is improving, pilot testing of all technologies will help to identify such implementation issues for troubleshooting.

##### Comparisons to In-Clinic Data

If data from more than one method of collection are going to be combined for analysis, then efforts should be made to harmonize across the data types. For instance, mobile phone versus in-clinic data variables should be comparable so that they can be compared at the stage of analyses, that is, they should have comparable questions, formats, and variables.

##### Participant Use

Iterative refinement of devices with user feedback on feasibility and acceptability is critical. Technologies will have variable participant acceptability depending on the setting and the type of technology. For example, use of an electronic fingerprinting technology to identify female sex workers in Zambia longitudinally within a mock HIV vaccine trial showed <50% uptake of the technology among women approached in the field (at their places of work) but >95% uptake within the clinic [11]. Pilot studies to assess technology acceptability by prospective participants before a trial are critical, as well as the understanding of what potentially modifiable barriers participants may face when taking up a technology. There may be particular concerns around confidentiality with the use of biometric devices.

#### Initial Successes May Not Be Sustained

Participation fatigue issues, described previously, can derail a study when initial success is not seen over the entire course of the study. Other issues that could result in the failure to maintain an early success include staff turnover, equipment failures (particularly when there is a lack of technical support), software upgrade issues that cause devices to become out of sync, or other changes to the overall system that affect the quality of the data to be collected.

#### Prepare for Some Percent of Failure

In designing a study, it is important to consider the effects of data that will be missed due to issues with the use of a chosen technology, especially when it is first implemented. Attempts to minimize the loss of data should be made throughout the study. Retraining during scheduled visits is very important. Identifying the challenges participants are experiencing in completing study procedures allows improvement of the user experience. Reminder strategies and plans to fit the mobile phone completion into the participant’s daily routine can be selected in consultation with the participant. Study staff should expect participants’ calls and provide support especially for those that default from study procedures. Regular check-ins (ie, telephonic contact and social networking applications) between study visits are important to keep the participants motivated for the daily survey submissions. It is also critical for a staff member to be trained to provide very technical information about mobile phone and Internet operation. For instance, users could unknowingly disable the Internet on their mobile phone and then not be able to enable it on their own.

#### Considerations for Study-Provided Devices

##### Devices May Be Manipulated by the Participants

In settings where mobile phone use may not be widespread, researchers may provide mobile phones as part of the participation in the study. Additionally, participants may be provided with the necessary airtime and data to perform study-related procedures. Providing a study phone can be advantageous. However, the disadvantage is that the user may misuse data and airtime provided for study purposes. To avoid participants using the study airtime for things unrelated to study, software can be installed to block data access to unrelated website that may affect the airtime for study purposes. Whereas safeguards can be put in place, technology-savvy people are often able to find a work-around.

For example, an app like “AppLock” can be used [52]. This App is password protected and Internet-enabled applications can be blocked by the data manager to ensure that uploaded data bundles are solely used for the project related application(s). Although participants may struggle to disable the blocking software, they would be able to reset the full phone, in which case the entire phone content would be erased. The phone would then have to be reset and the software reinstalled for study-related procedures to continue.

These problems can be addressed by providing sufficient data on a monthly basis for participants to meet both the research data collection and their personal needs. One concern for trials enrolling minors is that mobile data can be used by participants to access materials not desirable by their parents. Both the loss or damage concern and the data use issues can substantially impact budgets and need to be considered a priori.

##### Loss and Breakage Will Happen

Whereas researchers have found that provisioning mobile devices or data minutes is a successful recruitment strategy and method for incentivizing participants, it is important to remember that the devices will often be unintentionally lost or damaged by participants (5-10% replacement rate is common but may be as high as 30% depending on the study population; J Haberer, personal communication).

Better results can be achieved if participants take ownership in the research. One way to motivate safekeeping of the study phone is to let participants know upfront that they would keep the phone after study completion. This approach may not only increase the likelihood that the participants will give the study phone the extra care but may also increase their motivation to be adherent to all study procedures to the best of their abilities.

#### Data Integrity

As with any data-collection system, errors in the data will exist and need to be addressed by study staff. Technological solutions do offer some mechanisms to increase data quality such as automatic quality-control checks, which can be put in place to define the expected range for some data types. Additionally, participants may be shown a summary of their data before the data are officially submitted to the study, allowing them to catch obvious errors. However, technology can also introduce new data challenges. Duplicate data entries can be made in some systems, after which study staff need to establish which entry is valid for that particular day. Devices used to measure adherence, such as enhanced pill containers, could be opened by participants without medication consumption, leading to inaccurate data. Additionally, systems will respond differently to incomplete or partial data, such as when an Internet connection is lost mid-entry.

### Conclusions

#### How to Move Forward in HIV Vaccine and Biomedical Prevention Trials

When considering how to move toward integration of digital technologies into HIV prevention clinical trials and observational studies, three guiding messages emerged at the meeting. Taken together, they can offer an informed and realistic approach to the challenges posed by new technologies: change is hard, things change rapidly, and support is critical. An approach that prepares for slower uptake and acceptance of new technology but includes flexibility to respond to rapid pace of change within technologies and addresses the support needed for both scenarios may be most successful.

##### Change is Hard

Regulatory authorities may be hesitant to accept new technology such as electronic source data due to concerns about security of source data, loss of data, and access by site. Change is also hard at the clinical research site level, which will carry much of the additional administrative burden of managing risks with new technology. For example, in the initial stages of introducing electronic case report forms, paper copies may need to be maintained as a backup to ensure regulatory compliance.

##### Technology Changes Rapidly

While dealing with potentially slow acceptance of change brought by new technologies, the HIV prevention field will at the same time need flexibility to respond to the rapid pace of change within technologies. Future-proofing is often impossible in this setting. Sponsors and research organizations should expect rapid obsolescence and continuing need for upgrading, refreshing, and tailoring as technology changes. For example, when using mobile data applications, offline solutions can quickly become obsolete because 4G cellular networks now have dense coverage in some countries [53].

New technologies do not obviate the need for responsible data processes, including continuing review of consent, privacy, security, ownership, and transparency. These practices are particularly important in changing legal environments. For example, if biometric information is collected in clinical trials, data security will need to be reassessed if the government develops national fingerprint or facial recognition databases, which would allow identification of participants.

##### Support is Critical

Infrastructure issues were noted as an expected key element for technology support. Despite the growth in reliable power and Internet systems globally, infrastructure weaknesses still complicate computer system use. Power outages, Internet connectivity, and local-area network (LAN) issues all require interventions to improve utilization and acceptance of digital technologies. In a study using cloud-based fingerprint biometric tracking, Internet connectivity frustrated site staff and led to a relatively high failure rate for fingerprint matching (N Kiwanuka, personal communication).

Technical support for staff can be challenging where staff are clinically trained but not fluent in technology. In one setting, the majority of clinically qualified staff trained to use a tablet technology had not worked with a touchscreen before [11]. Trial participants also require support for use of new technologies, particularly mobile health apps.

Whereas on-site support and partnering with local equipment vendors for on-site service can be useful, several investigators felt that specialized technologies were better suited to a central-help desk. As additional new technologies become available, often with different support requirements and a limited expertise-base, the ability to successfully provide technical support may be the limiting factor in expanded use of new clinical trial technologies, not regulatory authorities or user acceptance.

### Summary

Digital technologies, especially if used in novel ways, provide a number of potential advantages to clinical research in trials related to HIV and AIDS and may greatly facilitate operations as well as data collection and analysis. These technologies may even allow answering questions that are not answerable with older technologies. However, they come with a variety of potential concerns for both the participants and the trial sponsors. The exact challenges and means for alleviation depend on the technology and on the population in which it is deployed. Nevertheless, in this guidance we aimed to summarize some common themes that are frequently encountered by researchers in this context and should be carefully considered before making a decision to include these technologies in their research. Finally, we encourage researchers to seek advice from organizations with past history and experience of applying digital technologies in HIV and AIDS clinical research.

## Abbreviations

AIDS: acquired immune deficiency syndrome
HIV: human immunodeficiency virus
JMIR: Journal of Medical Internet Research
LAN: local area network
PIN: personal identification number
RCT: randomized controlled trial
SMS: short message service
USSD: unstructured supplementary service data
